# HIV trends and disparities by gender and urban–rural residence among adolescents in sub-Saharan Africa

**DOI:** 10.1186/s12978-021-01118-7

**Published:** 2021-06-17

**Authors:** Réka Maulide Cane, Dessalegn Y. Melesse, Nkomba Kayeyi, Adom Manu, Yohannes Dibaba Wado, Aluisio Barros, Ties Boerma

**Affiliations:** 1 Women’s and Children’s Health Program, Instituto Nacional de Saúde, Ministério da Saúde, Maputo, Mozambique; 2 Institute for Global Public Health, University of Manitoba, Winnipeg, Canada; 3 Population Council, Lusaka, Zambia; 4 Department of Population, Family & Reproductive Health, School of Public Health, University of Ghana, Accra, Ghana; 5 African Population and Health Research Centre, Nairobi, Kenya; 6 Federal University of Pelotas, Pelotas, Brazil

**Keywords:** HIV prevalence, Trends, Disparities, Gender, Urban–rural, Adolescents, Sub-Saharan Africa

## Abstract

**Background:**

In sub-Saharan Africa HIV transmission is a major challenge in adolescents, especially among girls and those living in urban settings. Major international efforts have aimed at reducing sexual transmission of HIV. This analysis aims to assess the trends in HIV prevalence by gender in adolescents, as well as urban–rural disparities.

**Methods:**

HIV prevalence data at ages 15–19 years were obtained for 31 countries with a national survey since 2010 and for 23 countries with one survey circa 2005 and a recent survey circa 2015. Country medians and average annual rates of changes were used to summarize the trends for two subregions in sub-Saharan Africa, Eastern and Southern Africa and West and Central Africa, which largely correspond with higher and lower HIV prevalence countries. Data on HIV incidence at ages 15–24 and prevalence at 5–9 and 10–14 years were reviewed from 11 recent national surveys. Trends in urban–rural disparities in HIV prevalence and selected indicators of sexual and HIV testing behaviours were assessed for females and males 15–24 years, using the same surveys.

**Results:**

HIV prevalence among girls 15–19 years declined in eastern and Southern Africa from 5.7 to 2.6% during 2005–2015 (country median), corresponding with an average annual rate of reduction of 6.5% per year. Among boys, the median HIV prevalence declined from 2.1 to 1.2%. Changes were also observed in West and Central Africa where median HIV prevalence among girls decreased from 0.7 to 0.4% (average annual rate of reduction 5.9%), but not for boys (0.3%). Girl-boy differences at 10–14 years were small with a country median HIV of 1.0% and 1.3%, respectively. Urban females and males 15–24 had at least 1.5 times higher HIV prevalence than their rural counterparts in both subregions, and since the urban–rural declines were similar, the gaps persisted during 2005–2015.

**Conclusions:**

HIV prevalence among adolescents declined in almost all countries during the last decade, in both urban and rural settings. The urban–rural gap persisted and HIV transmission to girls, but not boys, is still a major challenge in Eastern and Southern African countries.

**Supplementary Information:**

The online version contains supplementary material available at 10.1186/s12978-021-01118-7.

## Background

High levels of HIV infection among adolescents, especially girls, have been a major concern in sub-Saharan Africa, where about 80% of the world’s HIV-infected adolescents live [1, 2]. Adolescent girls and young women aged 15–24 years accounted for one in five new HIV infections, despite being just 10% of the population of sub-Saharan Africa [3]. UNAIDS estimated that, between 2010 and 2017, there was a 25% decline in new HIV infections among girls aged 10–19 years in Eastern and Southern Africa, but no decline in West and Central Africa. A recent analysis of pooled data from longitudinal community studies in Eastern and Southern Africa showed that adolescent girls had 5.9 and 3.2 times higher HIV incidence than adolescent boys during 2005–2015, respectively, with only limited evidence of a decline over time [4].

Several global and national initiatives have called for greater attention to adolescent girls and HIV, often with ambitious targets. The ‘All In to End Adolescent AIDS’ initiative, led by UNICEF and other global agencies, aims to reduce HIV infections among adolescents 10–19 years by 75% by 2020 and end the AIDS epidemic among adolescents by 2030 [5]. The DREAMS partnership, launched in 2014, in ten countries in sub-Saharan Africa aims to bring down adolescent and young women new infections by 40% in a 2-year period [6].

The HIV epidemic in sub-Saharan Africa is characterized by large differences between countries and subregions, and adolescents are no exception [7]. Understanding local epidemiology within each country is essential to effectively target HIV prevention efforts [8]. This includes consideration of distal socioeconomic and residential characteristics as well as biological and behavioural factors that affect HIV transmission [9, 10]. Within countries, HIV epidemiology tends to vary by socioeconomic and geographic characteristics [11]. National surveys often show higher HIV prevalence among adolescents and adults with more education, living in wealthier households or in urban areas compared to less educated, poorer and rural residents [12–15]. These inequality patterns differ from those observed for many other infectious diseases and for reproductive, maternal and child health but may change if urban, wealthier and more educated populations are quicker to adopt interventions or behavioural changes. An analysis of seven countries in sub-Saharan Africa found limited evidence such changes among educated adolescents prior to 2013 [16].

This study aims to assess sex-specific trends in HIV incidence and prevalence in adolescents by urban–rural residence, using national surveys with HIV testing and modules on sexual behaviour. This assessment will focus on the big picture from about 2005 to the most recent data points until 2018 for two subregions of sub-Saharan Africa, Eastern and Southern Africa and West and Central Africa.

## Methods

The data were derived from national household surveys with HIV testing. We identified 77 surveys with HIV testing conducted in sub-Saharan Africa since 2003, including Demographic and Health Surveys (DHS), AIDS indicator Surveys (AIS) [17], Population HIV Indicator Assessment (PHIA) surveys [18] and specific national surveys (see Additional file 1). All surveys are nationally representative household surveys and included the collection of blood samples for HIV testing among female and male respondents 15–49 years (DHS and AIS) or 0–64 years (PHIA). Response rates in the surveys were high, as the median HIV testing and interview response rates for girls and boys 15–19 years in DHS and AIS surveys were about 90%, with only one survey with response rates below 70% (South Africa 2016).

Testing procedures for HIV prevalent infection may differ but all survey protocols involved confirmatory testing of positive cases [17, 18]. Recent advances have made it possible to directly estimate HIV incidence in cross-sectional surveys, using an algorithm based on limited antigen (Lag) avidity assay, viral load, and ARV use [19]. These statistics were available for respondents 15–24 years in eight PHIA survey reports that reported age-disaggregated data and one national survey in South Africa. The numbers were too small for disaggregation by urban–rural residence and no trend assessment could be done.

All surveys used a two-stage cluster-based sampling design to select households within selected census enumeration areas or equivalent unit. For the DHS and AIS surveys, conducted between 2003 and 2018, data sets are in the public domain and standardized indicators are available. The 11 PHIA surveys were conducted from 2015 but only four surveys data sets were available in the public domain at the time of our analyses (Eswatini, Malawi, Tanzania, and Zambia). We extracted information from the PHIA national reports for selected indicators if no primary data were available. Kenya, Nigeria, South Africa and Eswatini conducted other types of national HIV surveys for which only national reports could be accessed.

We focused on the assessment of trends in countries with surveys before and after 2010 (the most recent survey). We included Burkina Faso with its most recent survey in 2010 and Burundi with its first survey in 2010. We classified the countries into two geographic subregions, Eastern and Southern Africa, and West and Central Africa. We included Burundi and Rwanda in the West and Central Africa subregion because of their proximity to Central Africa and their greater HIV epidemiological similarity with this subregion (see Additional file 1). Trend data were available for 23 countries, with a median year of the first survey of 2005 and the second of 2014.

Subregional levels and trends were summarized with country medians and the annual average rate of change in HIV prevalence between the first (p1, referring to HIV prevalence at time t_1_) and last survey (p2, HIV prevalence at time t_2_), defined as the average relative percent change per year in HIV prevalence(ln(p_2_/p_1_)/(t_2_ − t_1_)). All analyses were conducted in Stata version 15 (StataCorp. 2017. Stata Statistical Software: Release 15. College Station, TX: StataCorp LLC) and MS Excel.

We tested for statistical significance of indicator trends over time by estimating the level of significance in the proportions’ differences using a formula developed by Altman and Bland [20]. We used 95% confidence intervals based on the Taylor linearization method used in DHS surveys and the jackknife repeated replication used for the PHIA surveys. The differences between the two methods are small in our case, as has been reviewed extensively elsewhere [21, 22].

HIV prevalence among adolescents has been used as a proxy measure for population incidence as the majority of infections represent recent sexually acquired infections and direct measurement of incidence was limited [23]. Even with HIV incidence measurement available, data are also needed to assess the distribution of prevalent infection in subpopulations. Because of the scale-up for antiretroviral therapy (ART) for children [24], prevalent adolescent HIV infection may include a rising number of pediatric infections who survive into adolescence, which affects the interpretation of adolescent prevalence statistics [25]. To obtain an idea of the size of this effect, we also examined HIV prevalence rates at ages 5–9 and 10–14 years, when sexual transmission is likely to be low, from recent PHIA surveys.

Even though our primary interest is in adolescents 15–19 years, we used data for the age group 15–24 years to disaggregate by urban–rural residence, to reduce sampling error. The correlation between HIV prevalence at ages 15–19 and 15–24 years was high for both females and males (Pearson’s correlation coefficient r > 0.90) (see Additional file 2). Urban–rural breakdowns were not available for the most recent surveys in Eswatini, Kenya and South Africa.

Urban–rural residence may affect HIV transmission through a set of proximate determinants which are behavioural in nature with a proven effect on the biological components of transmission such as risk of exposure and transmission efficiency [10, 26]. We examined trends in selected indicators of the proximate determinants, based on the availability of comparable time trend data from DHS, in urban–rural populations, disaggregated by gender:

- Sexual initiation: percentage of women/men 18–24 years who initiated sex before age 18
- Premarital sexual activity: percentage of young never married women/men age 15–24 who had sex in the last 12 months of all young single women/men surveyed among respondents 15–24 years
- Multiple partnerships: percentage of young women/men age 15–24 who had sex with more than one partner in the 12 months preceding the survey among women/men who had sexual intercourse in the 12 months preceding the survey
- Condom use: percentage of young never married women/men age 15–24 who used a condom at last sex, of all young single sexually active women/men surveyed
- HIV testing: percent who received an HIV test in the last 12 months among sexually active respondents 15–24 years.

## Results

### HIV prevalence at 15–19 years

In the 12 countries Eastern and Southern Africa, the median adolescent HIV prevalence in countries with a national survey conducted since 2010, was 3.3% for girls and 1.4% for boys 15–19 years old. HIV prevalence in the Southern African countries was highest and above 5% in four countries (Eswatini, Mozambique, South Africa, Lesotho). At the lower end, Ethiopia was the only country with HIV prevalence among girls below 1%. In West and Central Africa, the median of 19 countries was 0.7% for girls and 0.3% for boys with prevalence just above 1.0% in five countries (Sierra Leone, Gabon, Cameroon, Chad and Guinea) (Table 1 and Additional File 3).

**Table 1:** HIV prevalence among girls and boys 15–19 years, by subregion (with p-value for difference of proportions test trend over time)

Country	Survey year		Girls, 15–19 years			Boys, 15–19 years		
	First	Last	First	Last	p-value	First	Last	p-value
Eastern & Southern Africa								
Eswatini	2007	2017	10.1	7.2	0.03	1.9	3.9	0.59
Ethiopia	2005	2016	0.7	0.4	0.39	0.1	0.0	0.52
Kenya	2003	2018	3.0	1.2		0.4	0.5	
Lesotho	2004	2017	7.8	5.7	0.13	2.3	2.8	1.30
Malawi	2004	2016	3.7	2.0	0.09	0.4	0.9	1.28
Mozambique	2009	2015	7.1	6.5	0.71	2.7	1.5	0.15
Namibia		2017		4.7			2.7	
South Africa	2008	2016	6.7	5.9		2.5	4.1	
Tanzania	2004	2017	2.1	1.0	0.03	2.1	0.4	0.00
Uganda	2005	2017	2.6	1.8	0.12	0.3	0.5	1.33
Zambia	2007	2018	5.7	2.6	0	3.6	1.2	0.00
Zimbabwe	2006	2016	6.2	3.9	0.01	3.1	3.2	1.10
Median all		2017		3.3			1.4	
Median (trend)	2005	2017	5.7	2.6		2.1	1.2	
West & Central Africa								
Angola		2016		0.8			0.6	
Burkina Faso	2003	2010	0.9	0.1	0.04	0.7	0.4	0.54
Burundi	2010	2017	0.2	0.1	0.55	0.3	0.0	0.14
Cameroon	2004	2018	2.1	1.2		0.6	0.2	
Chad		2015		1.2			0.4	
Cote d'Ivoire	2005	2018	0.4	0.6		0.2	0.4	1.35
DR Congo	2007	2014	0.7	0.7	1	1.7	0.2	0.16
Gabon		2012		1.5			0.4	
Gambia		2013		0.4			0.3	
Ghana	2003	2014	0.5	0.3	0.55	0.2	0.2	1.00
Guinea	2005	2018	0.9	1.2	0.58	0.5	0.9	1.36
Liberia	2007	2013	1.3	0.2	0.03	0.4	1.0	1.36
Mali	2006	2013	0.6	0.8	0.67	0.7	0.3	0.36
Niger	2006	2012	0.0	0.0		0.0	0.0	
Nigeria		2018		0.7			0.1	
Rwanda	2005	2015	0.7	0.9	0.72	0.3	0.3	1.00
Senegal	2005	2017	0.2	0.0	0.08	0.0	0.0	1.00
Sierra Leone	2008	2013	1.3	1.5	0.75	0.0	0.7	0.31
Togo		2014		0.4			0.1	1.35
Median all		2014		0.7			0.3	
Median (trend)	2005	2014	0.7	0.5		0.3	0.3	

*n/a* not available

**Fig. 1:**
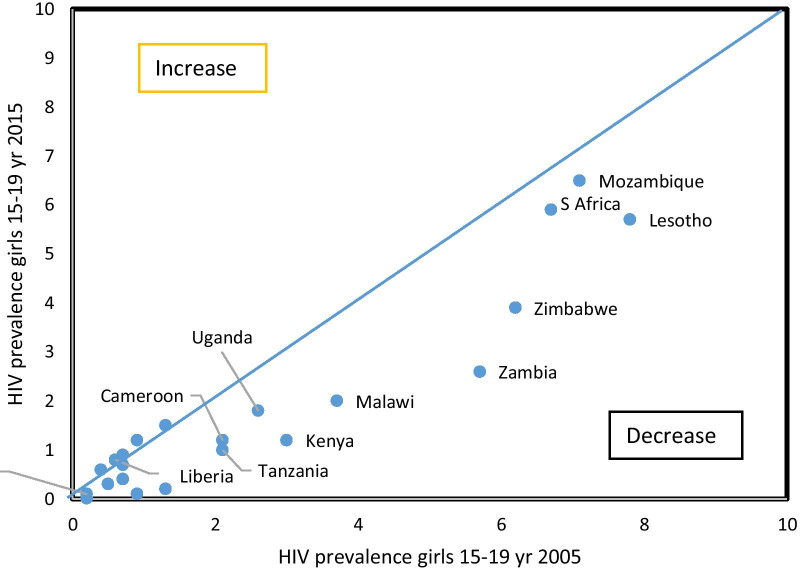
HIV prevalence trend among individual countries, surveys conducted circa 2005 and circa 2015

Among girls in the 11 countries in Eastern and Southern Africa with two data points, the median HIV prevalence decreased from 5.7 to 2.6% during 2005–2017, corresponding to an average annual rate of reduction of 6.5%, with reductions observed in all countries (Fig. 1). The difference between the two surveys was statistically significant at the 5% level in Eswatini, Malawi, Tanzania, Zambia and Zimbabwe.

In the 13 countries in West and Central Africa with two data points, median HIV prevalence among girls decreased from 0.7 to 0.3% (average annual rate of reduction 5.9%), with Liberia and Burkina Faso having the largest declines (p < 0.05).

Among boys, the median HIV prevalence declined from 2.1 to 1.2% in Eastern and Southern Africa. The average annual rate of reduction exceeded 10% in Kenya, Tanzania and Zambia, but in five countries no declines were observed. The difference between the two surveys was statistically significant at the 5% level in Tanzania and Zambia. In West and Central Africa, HIV prevalence remained low (country median of 0.3% in first and last survey).

### Early adolescence

**Fig. 2:**
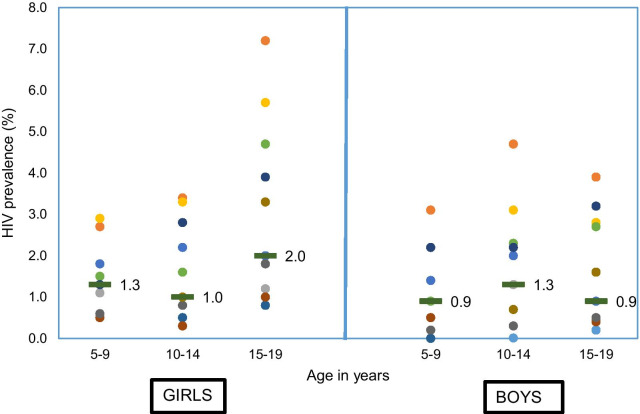
HIV prevalence at ages 5–9, 10–14 and 15–19 years, PHIA surveys and South Africa national survey conducted 2015 or later (dash indicates country median, dots represent country values)

Median HIV prevalence at 10–14 years was 1.0% for girls and 1.3% for boys among 10 countries with recent surveys (10 in Eastern and Southern Africa and Cameroon) (Fig. 2 and Additional file 4). The girl-boy differences were small (less than 1%), as expected in the case of predominance of pediatric HIV infections surviving into older adolescence, with the exception of Eswatini where boys (4.7%; 95% CI 2.8–6.5%) had considerably higher prevalence than girls (3.4%; 95% CI 1.8–5.1%). This difference, however, was not statistically significant. HIV prevalence at 5–9 years was similar to that at age 10–14 years in all countries, with a country median of 1.3% for girls and 0.9% for boys.

At ages 15–19 years, HIV prevalence was higher than at 10–14 years in nine of the 11 countries for girls (median 1.1% higher). The difference was statistically significant at the 5% level in six of the eight countries with sufficient data: Eswatini, Lesotho, Namibia, Tanzania, Uganda and Zambia. For boys, the differences between 15–19 and 10–14 years were small and not statistically significant in all countries.

### HIV incidence

In all nine countries with data, female HIV incidence at 15–24 years was considerably higher than for males (median absolute difference 0.9%). Among females, eight of nine countries had annual HIV incidence of 0.5% or higher. The three southernmost countries of sub-Saharan Africa (Eswatini, South Africa and Lesotho) stood out with HIV incidence rates of about 1.5% (Additional file 5). Among males 15–24 years, only Eswatini and South Africa had an annual incidence of 0.5%. All other surveys registered HIV incidence of 0.2% or lower, indicating generally low HIV transmission sampling errors for incidence data were large and no attempt was made to disaggregate by urban–rural residence or obtain estimates for adolescents 15–19 years.

### Urban–rural disparities: HIV prevalence

**Fig. 3:**
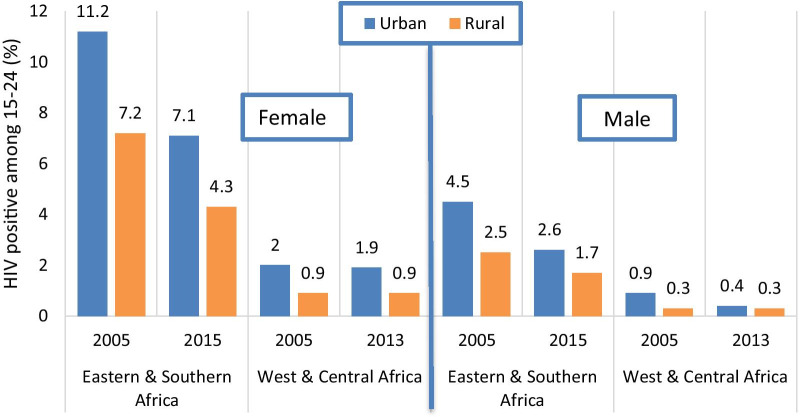
HIV prevalence among females and males 15–24 years by urban–rural residence, median of higher and lower prevalence country groups, in surveys conducted circa 2005 and circa 2015

Among eight countries with two surveys in Eastern and Southern Africa, the country median HIV prevalence at 15–24 years declined from 11.2 to 7.1% for urban females, at an annual rate of reduction of 4.6% (Fig. 3). Rural female HIV prevalence also declined from 7.2 to 4.3%, at a similar annual rate of reduction (5.2%). All countries experienced urban and rural declines in HIV prevalence which only in Zimbabwe (both urban and rural), Malawi and Cameroon (rural only), and Zambia (urban only) reached statistical significance at the 5% level (Additional file 6).

For males 15–24 years in Eastern and Southern Africa, urban and rural HIV prevalence declined from 4.5 to 2.6% and from 2.5 to 1.7% respectively. This corresponds with similar average annual rates of reduction as for females (5.7% and 4.2% for urban and rural, respectively).

Among 13 countries in West and Central Africa, there were no major changes over time for urban and rural females and for rural males. The only reduction was observed for urban males (from 0.9 to 0.4%), driven by a decline in six countries.

As a result, the median urban–rural HIV prevalence ratios remained similar over time and were 1.6 and 2.1 for females, and 1.5 and 1.3 for males, in the most recent surveys, in Eastern and Southern Africa and West & Central Africa respectively.

### Urban–rural disparities: behaviours

Table 2 summarizes the urban–rural trends in adolescent and young adult sexual behaviour and HIV testing during 2006/07 and 2014/15 by sex and subregion (country data—Additional File 7). In general, the country median values for the sexual activity indicators of age at first sex, multiple partnerships and premarital sex did not change much between the first and last surveys.

**Table 2:** Selected behavioural indicators among women and men 15–24 years by subregion, urban and rural residence, national surveys (median values, percent, countries)

	Urban		Rural		Urban–rural ratio	
	2006/7	2014/5	2006/7	2014/5	2006/7	2014/5
**Eastern and Southern Africa**						
Female						
Sex before age 18	48.4	46.8	60.9	60.6	0.79	0.77
Premarital sex	34.5	37.7	27.2	30.7	1.27	1.23
Multiple partners in last year	4.9	5.5	3.0	3.3	1.63	1.67
Condom use at last non-marital sex	54.3	64.4	32.8	46.0	1.66	1.40
HIV tested in last year	31.9	61.7	16.6	52.3	1.92	1.18
Male						
Sex before age 18	52.1	51.7	54.5	55.4	0.96	0.93
Premarital sex	42.5	48.7	38.7	44.8	1.10	1.09
Multiple partners in last year	25.6	27.3	22.9	22.9	1.12	1.19
Condom use at last premarital sex	72.3	78.1	45.6	65.7	1.59	1.19
HIV tested in last year	18.6	47.1	11.2	32.8	1.66	1.44
**West and Central Africa**						
Female						
Sex before age 18	44.7	44.8	66.7	66.8	0.67	0.67
Premarital sex	33.4	29.4	31.0	21.3	1.08	1.38
Multiple partners in last year	4.6	4.2	2.7	3.1	1.70	1.35
Condom use at last non-marital sex	35.8	37.7	16.4	18.5	2.18	2.04
HIV tested in last year	7.8	19.8	1.5	15.2	5.20	1.30
Male						
Sex before age 18	36.5	28.1	29.0	24.8	1.26	1.13
Premarital sex	31.9	35.4	27.2	23.6	1.17	1.50
Multiple partners in last year	22.3	25.3	22.5	22.5	0.99	1.12
Condom use at last premarital sex	57.0	62.2	27.9	43.4	2.04	1.43
HIV tested in last year	4.4	9.3	2.1	5.0	2.10	1.86

Sex before age 18 was and remained more common in rural girls than in urban girls in both subregions, with no change over time. Also, among boys sex before 18 was nearly equally common in the most recent surveys among urban and rural boys in both subregions, except in West and Central Africa where there was due to a faster decline in rural boys.

Premarital sexual activity in the last 12 months was higher among urban boys and girls in Eastern and Southern Africa and increased in both urban and rural populations, with little change of the urban rural ratio over time. In West and Central Africa, premarital sex was also more common in urban settings and the urban rural difference increased due to faster decline in the rural populations.

Multiple partnerships in the last year, which were infrequently reported by females, were more common among urban women in both subregions with little change over time. Among males 15–24, the urban rural differences were small and did not change over time.

Condom use at premarital sex among females and males was higher in urban than rural areas, and higher in Eastern and Southern Africa, with countries more affected by HIV, than in the West and Central Africa. There was an increase among urban and rural populations in the period between the two surveys, which was greater in rural populations, reducing the urban–rural gap. Urban condom use however remained considerably higher than rural use, especially in West and Central Africa.

HIV testing in the last 12 months among sexually active female and male respondents 15–24 years increased dramatically in both urban and rural settings in Eastern and Southern Africa. The urban rural gap reduced considerably due to a faster increase in the rural population. In West and Central Africa, HIV testing also increased among all groups, albeit still at much lower levels than Eastern and Southern Africa, faster in rural than urban settings.

## Discussion

HIV prevalence among adolescent girls 15–19 years decreased in both subregions of sub-Saharan Africa between 2005 and 2015, by as much as 54% in Eastern and Southern Africa where prevalence is higher and by 43% in West and Central Africa where prevalence is generally 1% or lower. Among boys 15–19 years, HIV prevalence is generally less than half the levels of girls, the median prevalence declined in Eastern and Southern Africa by 43% but remained low in West and Central Africa (0.3%). These findings confirm the presence of a positive trend towards lower HIV transmission in many countries.

The prevalence trends among girls 15–19 years are likely to present an underestimate of the reductions in HIV transmission due to sexual behaviour. Prior to the introduction of antiretroviral therapy (ART) in children, only a small proportion of adolescents would have acquired HIV from their mothers, as most would have died before reaching age 15. Most adolescent infections would be sexually transmitted, almost exclusively to girls due to partnerships with infected older men, as their male peers have HIV prevalence close to zero in many countries. The comparison of HIV prevalence at 15–19 years with earlier age groups gives an indication of the size of the effect of increasing coverage with ART in children with vertically transmitted HIV. Prevalence was about 1% among children 5–9 and 10–14 years in the higher prevalence countries with little negligible girl-boy differences in both age groups and a much higher prevalence at 15–19 only among girls.

HIV incidence results in recent surveys are consistent with this finding. Annual incidence among females 15–24 years was at least 0.5 per 100 per year in most countries with higher prevalence, while male levels are close to zero. Unfortunately, incidence measurement in surveys is still affected by sample size limitations for this relatively rare event, and no estimates for 15–19 years or time trends are available.

These results confirm the importance of sexual transmission among girls and not among boys, given the difference between 10–14 years and 15–19 years prevalence among girls but not boys, and the large sex differences in HIV prevalence at 15–19 years which are not expected as a result of only vertical transmission. Elsewhere, maternal transmission rather than sexual transmission was also considered a likely explanation for the higher prevalence among adolescent orphans 15–17 years old compared to non-orphaned peers [27].

In virtually all countries, urban and rural HIV prevalence among adolescents and young people 15–24 years declined at a similar pace, leaving the gap unchanged. Urban residents however are however still disadvantaged with higher HIV with urban rural ratios among females of 1.6 and 2.1 in Eastern and Southern Africa and West and Central Africa, respectively.

Self-reported indicators of behavioural risk did not help explain this urban disadvantage. Sexual risk behaviours as measured by sexual initiation before age 18, premarital sexual activity and multiple partnership among sexually active adolescents and young people, were remarkably similar in the 23 countries with two surveys conducted around 2005/2006 and 2015/2016. The urban rural gaps remained similar. There was however an increase in reported condom use at premarital sex in both higher and lower prevalence countries, and a major increase in HIV testing rates among adolescents and young adults of both sexes in urban and rural respondents. The urban–rural gap reduced, as rural residents experienced a faster increase. The increase in condom use in both urban and rural adolescents is encouraging, even though there are concerns about the shift in emphasis on long term methods in family planning programs leading to less emphasis on condoms which is the only method that protects against pregnancies and sexually transmitted infections [28]. Even though there are substantial knowledge gaps about what works best, the provision of sexuality education and sexual and reproductive health services. increasing awareness, acceptance, and support for youth-friendly sexual and reproductive health education and services and addressing gender inequalities have been singled out as priority interventions [29].

Our study has several limitations. Our focus on subregional inequalities and trends ignored the substantial variation between countries with the subregions. The most prominent outlier was Ethiopia within Eastern and Southern Africa with much lower HIV prevalence than all other countries in this subregion, but also differences between Eastern and Southern African countries were large in terms of levels and trends. Also, within West and Central Africa there were important differences between countries, but it is beyond the scope of this paper to discuss these individual trends. The urban–rural analyses were based on ages 15–24 years for disparity analysis to reduce sampling errors. We however showed that HIV prevalence at 15–24 years was highly correlated with 15–19 years and is likely to be indicative of urban–rural differences at ages 15–19 years. All other indicators focused on adolescent HIV and related risk behaviours through a combination of current status data and recall by survey respondents 15–24 years.

Regarding behavioural indicators, we considered only multi-country and subregional trends for a limited number of indicators with comparable data. The urban–rural differences in HIV and behaviours appeared weakly correlated. In fact, several behavioural indicators in this study would suggest higher HIV risks for rural than urban adolescents, which suggests that other proximate factors are likely to play a role. These may include risk related to age mixing (younger girls having sex with older men), concurrent partnerships and sexual networks, other sexually transmitted infections, and male circumcision [30–34]. There is some evidence from surveys of increases in male circumcision rates in multiple countries, and more so in urban areas [35]. In addition, sexual behaviour reporting is subject to multiple biases, of which underreporting related to stigma or desirability bias is the most problematic, especially by girls and young women [36–39]. The consistency of the results between consecutive surveys in the same country was however remarkable, suggesting no major change in bias over time. In addition, our analysis did not consider within urban inequalities, especially the HIV risk among the urban poor [40]. This issue needs more attention in future research.

## Conclusion

Our main objective was to assess the general sex-specific HIV trends in urban and rural adolescents in two major subregions of sub-Saharan Africa. HIV prevalence data suggest that HIV transmission among adolescents 15–19 years have reduced considerably in the past decade in both Eastern and Southern Africa and West and Central Africa, in spite of increasing survival of children who acquired HIV from their mothers into adolescence. This may be due to increased testing and ART and increased condom use. However, incidence and prevalence issue indicate that sexual transmission is still a major issue for adolescent girls. Adolescent and young adult HIV prevalence was still 1.5 to two times higher in urban populations compared to rural population in both subregions, with little change over time. Urban adolescent girls remain at greatest risk of HIV especially in higher prevalence countries and need to be targeted in prevention programs.

## Supplementary Information

**Additional file1.** National surveys with HIV testing with year of completion, xls, 33-countries table checklist.

**Additional file2.** Correlation between HIV prevalence at ages 15–19 and 15–24 by gender, xls, 02 figures for females (A) and males (B).

**Additional file3.** HIV prevalence 15–19-year-old girls and boys with confidence intervals, by country.

**Additional file4.** HIV Incidence at ages 15–24 by gender, xls, 04 tables and 01 figure.

**Additional file5.** HIV prevalence at 5–9, 10–14 and 15–19 years, xls.

**Additional file6.** Urban–rural HIV prevalence trends in adolescents by sex, 15–24 years, xls, 02 tables.

**Additional file7.** Selected behavioral indicators among women, 15–24 years, grouped by HIV prevalence, in urban and rural residence, national surveys (media percent, countries), xls, 02 tables.

## Data Availability

Reasonable requests can be made to access the data analyzed in this study from the corresponding author.

## Abbreviations

AIDS: Acquired Immune Deficiency Syndrome
ART: Antiretroviral Therapy
ARV: Antiretroviral (drug)
AIS: AIDS Indicator Survey
DHS: Demographic and Health Survey
DREAMS: Determined, Resilient, Empowered, AIDS-free, Mentored, and Safe
HIV: Human Immunodeficiency Virus
PHIA: Population HIV Indicator Assessment
UNAIDS: Joint United Nations Program on HIV/AIDS
UNICEF: The United Nations Children's Fund
